# Liquid-Crystalline Order in the Phosphorus-Containing DenDrimers

**DOI:** 10.3390/molecules27238214

**Published:** 2022-11-25

**Authors:** Victor Furer, Alexandr Vandyukov, Jean-Pierre Majoral, Anne-Marie Caminade, Valery Kovalenko

**Affiliations:** 1 Department of Physics, Electrical Engineering and Automation, Kazan State Architect and Civil Engineering University, 1 Zelenaya Str., 420043 Kazan, Russia; 2 A.E. Arbuzov Institute of Organic and Physical Chemistry, RAS, 8 Arbuzov Str., 420000 Kazan, Russia; 3 Laboratorie de Chimie de Coordination, CNRS, 205 Route de Narbonne, CEDEX 4, 31077 Toulouse, France

**Keywords:** phosphorus-containing dendrimers, IR spectra, dipole moment, microscopy

## Abstract

The structure of phosphorus-containing dendrimers has been studied by IR spectroscopy and optical polarization microscopy. The repeating units of dendrimer molecules are mesogens. This property arises from the conjugation of the aromatic ring and the hydrazone group. An analysis of the IR spectra showed that, with an increase in the generation number, the width of the stretching vibration bands ν(PN) and ν(PO) increases. Difficulties in packing molecules of higher generations cause conformational diversity. The shape of the dendrimer molecules was determined by analyzing the increments of dipole moments. Additionally, the modeling of the stacking of repeating links was performed. The spherical model of molecules does not satisfy the experimental dipole moments of the dendrimers. The flat disk model is more suitable for explaining step changes in dipole moments. The liquid-crystalline ordering of dendrimers under the action of applied pressure was found. With simultaneous heating and uniaxial compression, optical anisotropy appears in dendrimers. It is associated with the formation of liquid-crystalline order. However, a thermodynamically stable liquid-crystalline phase is not formed in this case. Dendrimers most likely have disk-shaped molecules.

## 1. Introduction

Dendrimer molecules can have different spatial shapes. In particular, most dendrimer molecules are spherical [1]. As a rule, these are conformationally mobile molecules that take the most compact form in space. Such are, for example, polyamidoamine dendrimers [2]. The molecule also takes on a spherical shape if there are many branches. Thus, dendrimers with a cyclotriphosphazene core have a spherical shape [3]. The repeating unit of such a dendrimer is sufficiently flexible.

If there are anisometrically rigid components, dendrimers can adopt a non-spherical shape [4]. Non-sphericity is accomplished because the individual dendron and repeating unit structures control how the dendrimer molecules take their shape.

Because of the variety of dendritic structures, some have mesogenic groups in repeating units or terminal groups and have liquid-crystalline properties. Therefore, studies of the mesomorphism of dendrimers are currently being actively conducted [5]. Some dendrimers acquire liquid-crystalline properties only under simultaneous exposure to heat and pressure [6].

The repeating units of phosphorus-containing dendrimers include aromatic rings conjugated with hydrazone groups [7]. This fragment has an elongated shape and is a typical mesogen. It would be interesting to determine the shape of the molecules of phosphorus-containing dendrimers. It is important to determine the conditions for the liquid-crystalline ordering of these dendrimers.

For some linear polymers, the liquid-crystalline state is formed under pressure. Such pressure-induced mesomorphism was found for 4-methoxybenzoic and 4-ethoxybenzoic acids, but similar studies have not been undertaken for dendrimers [8]. It is interesting to study the liquid-crystalline ordering in phosphorus-containing dendrimers. Polarizing microscopy is the most convenient method for observing liquid-crystalline order. We will attempt to obtain the liquid-crystalline state of phosphorus-containing dendrimers. 

## 2. Results

### 2.1. Optical Microscopy

Observations made with a polarizing microscope showed that phosphorus-containing dendrimers are amorphous substances. Generations G’_0_ and G_1_ are crystalline, with melting points of 106° and 75 °C, respectively. When samples of dendrimers are heated to a temperature of 250 °C, the substance becomes liquid. The optical anisotropy characteristic of the mesophase is not detected.

Pressure and high temperatures lead to the appearance of an anisotropic mesophase in dendrimers. Under the action of pressure on dendrimer samples at high temperatures, a liquid crystal order arises. When the load is removed, the order disappears.

**Figure 1:**
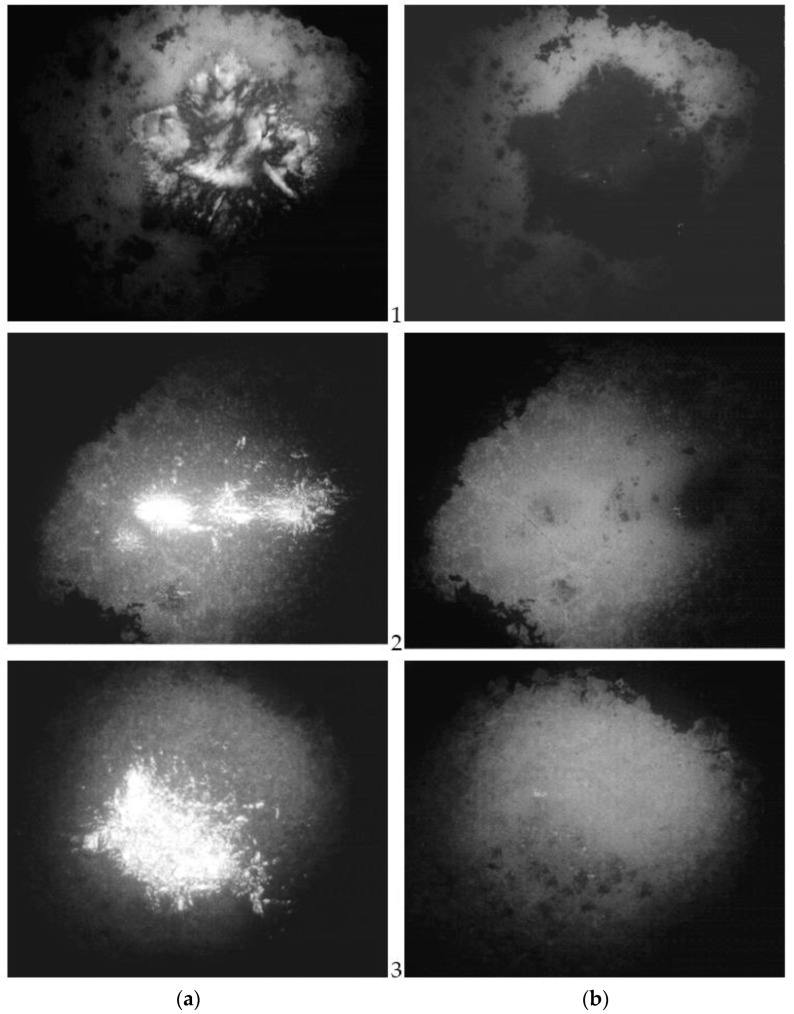
Micrographs of samples of phosphorus-containing dendrimers of generations G_2′_ (1), G_4′_ (2), and G_9′_ (3) obtained at a temperature of 250 °C; under pressure (**a**) and without pressure (**b**). The polarizer was oriented from east to west. The analyzer oriented the direction of oscillation from north to south.

The transitions observed for generations G’_1_–G’_10_ dendrimers to an anisotropic state were recorded using photographs. It can be seen that the texture disappears when the pressure is removed (Figure 1). An aromatic ring conjugated with a hydrazone group in the dendrimer is a typical mesogen.

Experiments with the simultaneous influence of temperature and pressure were conducted. The relationship between temperature, pressure, and generation number is established. The temperature at which liquid-crystalline order occurs increases for high-generation dendrimers.

This dependence is significant at temperatures below 120–140 °C, while at higher temperatures it is weak. The required pressure increases as the temperature of the sample decreases. It gradually decreases to a value of the order of 10 kg/cm^2^ at higher temperatures and depends little on the generation number. The liquid-crystalline order is fixed upon cooling and maintaining the applied pressure.

At temperatures around 300 °C, the destruction of dendrimers begins. The induced liquid crystal order is preserved due to interdendron “crosslinks”. It is fixed when the pressure is removed and the sample is cooled.

**Figure 2:**
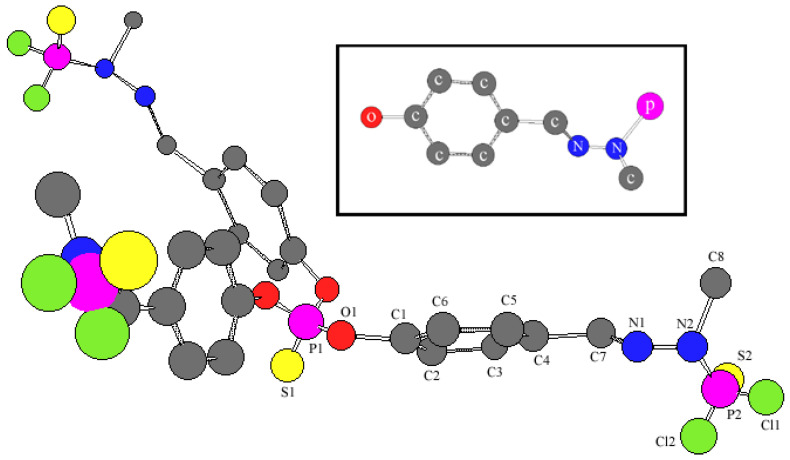
The first-generation dendrimer’s molecular structure [9]. The inset shows the fragment of the repeating unit that corresponds to it.

Planar repeating units of the dendrimer determine their ability to self-organize. Dendrons are packed into a disk-like structure (Figure 2). The presence of periodic branching does not facilitate the stacking of dendrons, like disks. For this reason, in addition to heating, the application of pressure is necessary. This creates conditions for a denser arrangement of repeating units due to conformational rearrangements.

### 2.2. Dipole Moments

An analysis of the dipole moments indicates the disk-like shape of the dendrimer molecules. The modeling of the stacking of repeating units is consistent with a similar shape of molecules. Dipole moments in linear polymers are known to determine the shape of molecules. The dipole moment of flexible-chain polymers is in the tens of debyes, while the dipole moment of rigid-chain polymers is in the thousands of debyes [10]. 

**Figure 3:**
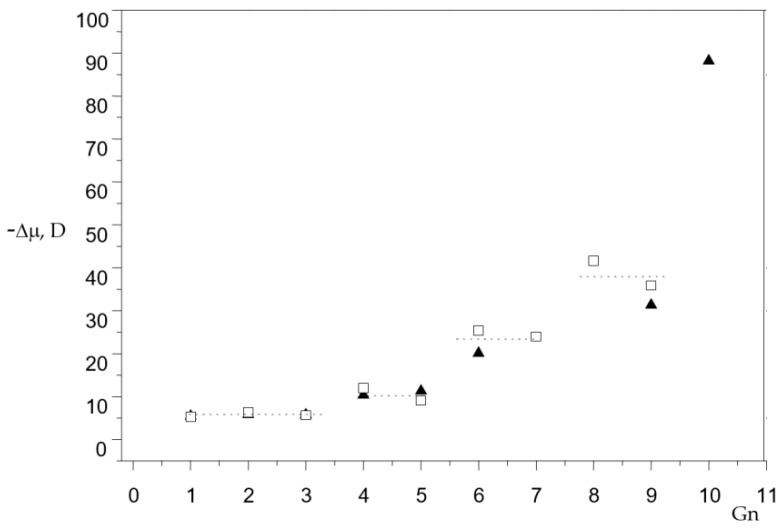
Dipole moments (D) are increased in steps based on the number of generations (^▲^ - dendrimers with terminal chlorine atoms, ^□^ - dendrimers with oxybenzaldehyde terminal groups).

Spherical dendrimers with low conformational mobility have a small dipole moment. The dipole moment of the phosphorus-containing dendrimer of the G’_11_ generation is 300 D [9]. To analyze the shape of dendrimers, let us consider how the dipole moment increases with an increase in the number of generations. One interesting feature is striking: the dipole moment for generations 1 to 3 increases by the same value for dendrimers with different end groups (Figure 3). This pattern persists for higher generations (Table 1).

**Figure 4:**
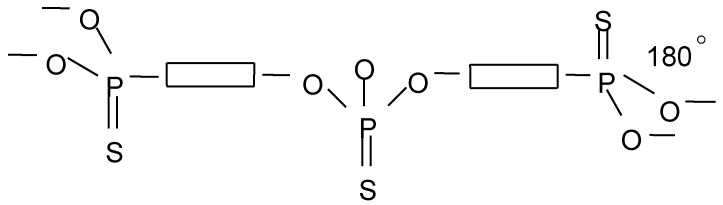
Probable conformations of the repeating unit in dendrimer molecules.

The spherical model of the molecule does not satisfy the experimental values of the dipole moments of dendrimers. The flat disk model is more suitable for explaining experimental data. The shape of the molecule depends on the orientation of the phenylenehydrazone fragments. The dipole moment of a dendrimer molecule depends on the parallel or antiparallel orientation of the polar groups (Figure 4).

In this case, the probability of complete compensation of the dipole moments of the individual groups is high. The increment of the dipole moment can be the same if the bond packing in the dendrons is the same for these generations. A change in packing leads to an increase in the dipole moment of the dendrimer.

A change in the shape of a dendrimer molecule leads to a change in its dipole moment. However, this cannot explain the observed large increase in dipole moments. Therefore, the most probable shape of the molecules of the dendrimer is a disk.

### 2.3. IR Spectroscopy

In dendrimers, internal rotation around PO and PN bonds is possible [11,12]. Therefore, attention should be paid to the change in the width of the stretching vibration bands of these bonds. The bandwidth in the IR spectra depends on the magnitude of internal stresses in macromolecules. An increase in the number of conformations is accompanied by an increase in the width of the bands in the IR spectra [13]. 

**Figure 5:**
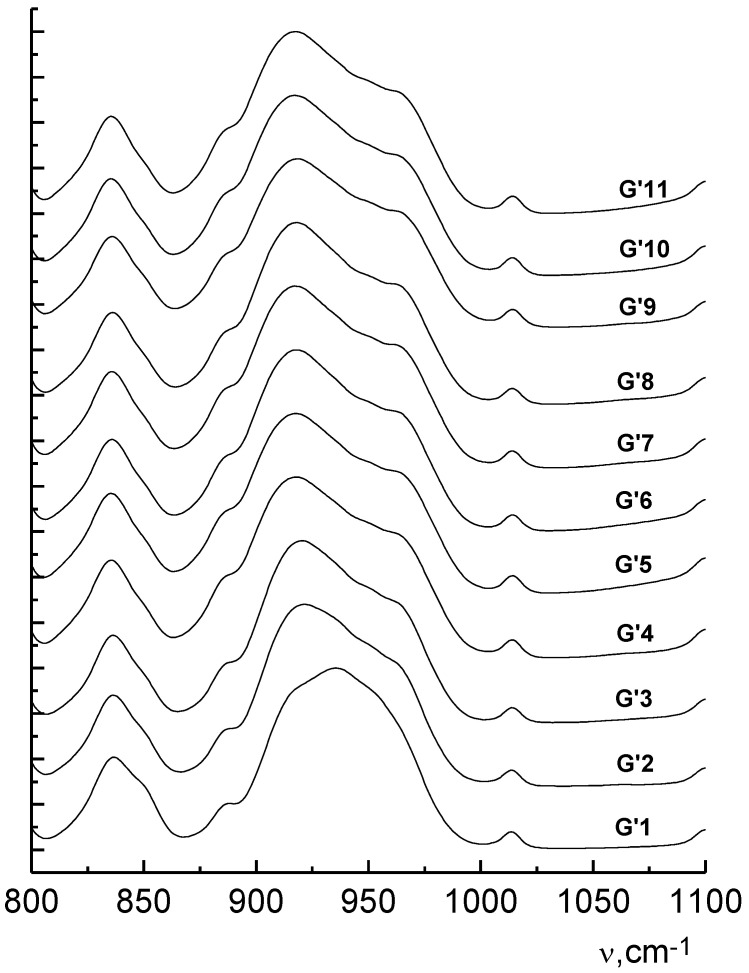
Modification of the contour of the ν(PO) and ν(PN) bands in the IR spectra of the generation series of phosphorus-containing dendrimers.

**Figure 6:**
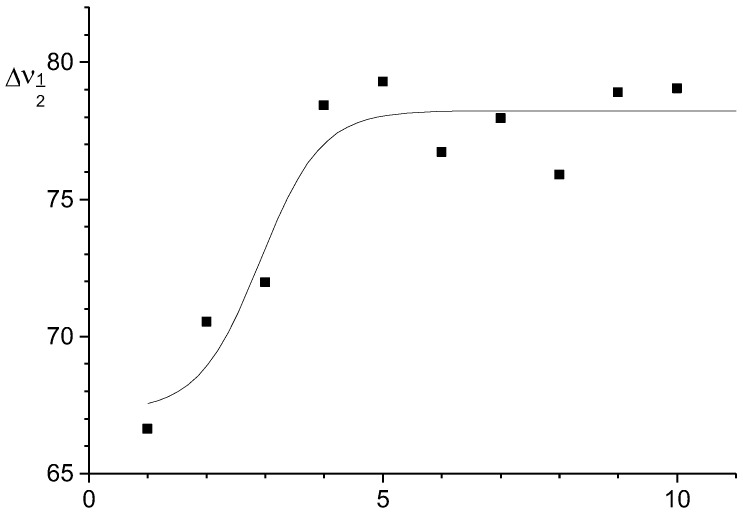
The dependence of the half-width of the absorption band stretching vibrations ν(PO) and ν(PN) in the IR spectra on the generation number G’_n_.

An analysis of the IR spectra showed that with an increase in the generation number, the width of the stretching vibration bands ν(PN) and ν(PO) increases (Figure 5 and Figure 6). This indicates the conformational diversity of these fragments. In the packing of higher generations, steric hindrances arise. They prevent the achievement of the most energetically favorable conformational states. This diversity leads to a corresponding broadening of the bands in the IR spectra. They are practically invisible in the Raman spectra.

## 3. Discussion

The liquid-crystalline order of dendrimers is similar to the mesomorphic order of linear polymers. In the case of dendrimers, this effect is determined by the shape and the cooperative intramolecular interaction of the dendrons in the molecule itself. Cooperative intermolecular interaction of fragments is realized in a linear polymer chain. 

The modeling of the stacking of molecules is consistent with their disk-like shape. Molecule sizes correspond to experimental data [14]. The experimentally measured diameter of the 10th-generation molecule, equal to 150 Å, is in good agreement with the calculated value for a flat stack of dendrons. 

Molecular dynamics calculations showed that the combination of “rigid” anisotropic fragments of the dendrimer and “nodes” of low conformational mobility determines the disk-like shape of the molecule [15]. It is interesting to note that the absence of such “hinges” in very rigid aromatic dendrons leads to the spherical shape of the molecules [15]. 

Our studies have shown that the molecules of phosphorus-containing dendrimers are disk-shaped. Anisotropic fragments and flexible bonds are required to realize the liquid-crystalline phase.

The observed phenomenon must be distinguished from piezo-optical effects or photoelasticity. The textures of the observed birefringence differ. The applied pressure for dendrimers is 2–3 orders of magnitude lower than for linear polymers.

A number of linear polymers containing anisotropic fragments were analyzed. A phenomenon similar to that observed in dendrimers was not found. Typical photoelastic patterns for polyethylene terephthalate were observed under a polarizing microscope at temperatures ranging from room temperature to 220 °C. Near the viscous-flowing state of the polymer, a similar texture is fixed. 

We have investigated rigid-chained, amorphous dendrimers that include phosphorus. It becomes apparent that they exhibit the ability to organize liquid-crystalline order under pressure. This essentially kinetic state is characterized by reproducibility. It is the limit of the classical thermodynamically stable liquid-crystalline phase. Of particular interest is the ability of dendrimer molecules to self-organize. This is determined by their disk shape.

The study of the mechanically induced liquid crystal order of dendrimers is just beginning. Its existence in other types of dendrimers remains to be seen. The potential practical possibilities of this effect for optical applications can already be assessed. 

We used this technique to study a number of generations of organosilicon dendrimers with very flexible mobile repeating units. However, no such effect was found. This is due to the isotropic, spherical shape of the molecules. Detailed temperature-dependent, wide-angle, and small-angle X-ray diffraction will be the subject of future research.

The use of phosphorus-containing dendrimers makes it possible to obtain sensitive, specific, and reusable sensors [16]. These dendrimers can be used as chemical and biological sensors. Fluorescent dendrimers are used for biological imaging.

## 4. Materials and Methods

**Figure 7:**
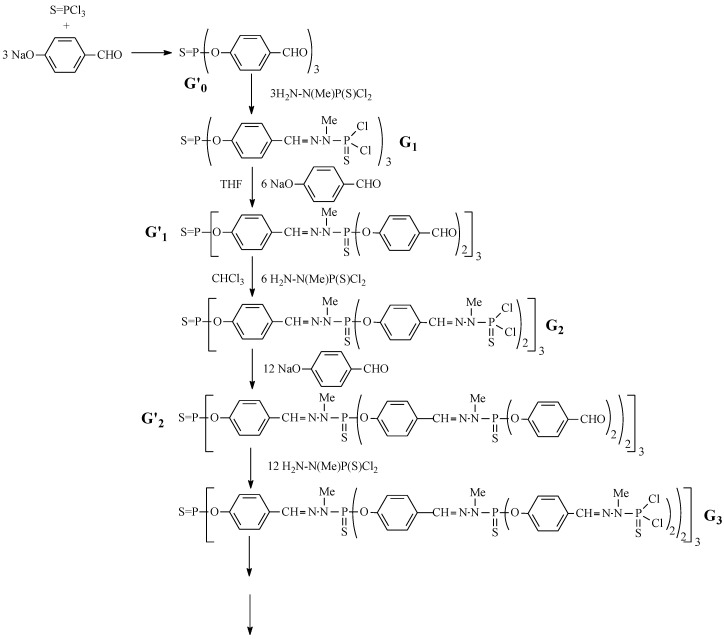
The sequence of reactions is repeated to produce 12th-generation dendrimers.

We have studied two series of dendrimers, Gn and G’n (Figure 7). 

The nucleophilic substitution reaction of 4-hydroxybenzaldehyde with P(S)Cl_3_ was the first step [7]. The second step is the Schiff reaction between the aldehyde fragments and the phosphorohydrazide. 

After condensation, the first-generation dendrimer, G_1_, is obtained. Repeating the reaction with the sodium salt of hydroxybenzaldehyde gives the second-generation G_2_ dendrimer. This process is repeated until the formation of the 12th generation of the dendrimer. Each step of the synthesis was monitored by ^31^P NMR (81.01 MHz in CDCl_3_, Table 2). 

The geometric parameters of G’_0_ and G_1_ molecules were determined by XRD [9]. The molecular structure of the generations is as follows: the trifunctional core S = P(O)_3_, repeating unit –C_6_H_4_−CH=N−N(CH_3_)−P(S)<, terminal groups of 4-oxibenzaldehyde fragments, –C_6_H_4_−CHO. 

The IR spectra of the dendrimers were recorded on a Bruker IFS-113v spectrophotometer (Bruker, Germany). 

To study the optical properties of dendrimers, an MIN-8 polarizing optical microscope was used. The magnification of the microscope was 31.

The temperature was measured on a Boetius heating table. Photographs of textures were obtained from a series of 10 generations of a dendrimer placed between glass plates and placed on a microscope heating stage. Experiments with increased pressure were conducted manually with a spatula. In addition, a special cuvette that exerts uniform pressure on the sample was used. The cuvette provided uniform heating of the sample throughout the entire volume. The base of the cuvette was attached to the heating table so that the hole in the base coincided with the hole on the heating table for better heat transfer.

## 5. Conclusions

Molecules of phosphorus-containing dendrimers of all generations have an anisometric structure of repeating units. Therefore, they can exhibit a liquid-crystalline order. This order is realized only under the simultaneous action of temperature and pressure. When heated without pressure, these substances remain amorphous. 

The observed order is due to anisotropic repeating units and the self-organization of dendrons. These structural features determine the association of disk-shaped dendrimer molecules.

By rapidly chilling the samples under pressure, the liquid-crystalline order can be fixed. This can also be achieved at very high temperatures using dendron crosslinking processes. The stable reproducibility of the phenomenon reflects the possibility of its practical applications. Dendrimers can be used as new optical temperature and pressure sensors. 

## Figures and Tables

**Table 1:** Analysis of dipole moments of molecules of phosphorus-containing dendrimers.

G_n_	Number of P=S Groups	μ, D (G_n_)	Δμ, D (G_n_)	μ, D (G_n_’) [9]	Δμ, D (G_n_’)
0	1	3.03	-	3.03	-
1	4	8.43	5.4	8.27	5.24
2	10	14.24	5.81	14.63	6.36
3	22	20.00	5.76	20.28	5.65
4	46	30.36	10.36	32.24	11.96
5	94	41.66	11.3	41.27	9.03
6	190	61.84	20.08	66.65	25.38
7	382	-	-	90.65	24.0
8	766	138.5	-	132.3	41.65
9	1534	169.8	31.3	168.2	35.9
10	3070	258.0	88.2	-	-
11	6172	-	-	328.0	-

**Table 2:** ^31^P NMR chemical shifts of phosphorus-containing dendrimers.

G_n_	δ(^31^P) ^1^												
	P_0_	P_1_	P_2_	P_3_	P_4_	P_5_	P_6_	P_7_	P_8_	P_9_	P_10_	P_11_	P_12_
G_1_	52.3	63.1	-	-	-	-	-	-	-	-	-	-	-
G’_1_	52.5	60.4	-	-	-	-	-	-	-	-	-	-	-
G_2_	52.6	62.0	63.2	-	-	-	-	-	-	-	-	-	-
G’_2_	52.7	62.2	60.6	-	-	-	-	-	-	-	-	-	-
G_3_	52.7	62.3	62.0	63.2	-	-	-	-	-	-	-	-	-
G’_3_	52.7	62.7	62.3	60.4	-	-	-	-	-	-	-	-	-
G_4_	52.5	62.7	62.3	62.0	63.1	-	-	-	-	-	-	-	-
G’_4_	52.5	62.9	62.7	62.3	60.4	-	-	-	-	-	-	-	-
G_5_	52.5	63.1	62.7	62.3	62.0	63.1	-	-	-	-	-	-	-
G’_5_	52.6	63.1	62.9	62.7	62.3	60.4	-	-	-	-	-	-	-
G_6_	52.6	63.1	62.9	62.7	62.3	62.0	63.1	-	-	-	-	-	-
G’_6_	52.5	61.7	61.7	61.7	61.7	61.5	60.0	-	-	-	-	-	-
G_7_	-	63.1	63.1	63.1	62.3	62.2	62.0	63.1	-	-	-	-	-
G’_7_	-	63.0	63.0	63.0	63.0	62.7	62.3	60.4	-	-	-	-	-
G_8_	-	63.1	63.1	63.1	63.1	62.6	62.3	62.1	63.1	-	-	-	-
G’_8_	-	62.9	62.9	62.9	62.9	62.9	62.7	62.3	60.4	-	-	-	-
G’_10_	-	63.0	63.0	63.0	63.0	63.0	63.0	63.0	62.8	62.4	60.4	-	-
G_11_	-	63.0	63.0	63.0	63.0	63.0	63.0	63.0	62.6	62.4	62.0	-	-
G’_11_	-	63.0	63.0	63.0	63.0	63.0	63.0	63.0	62.8	62.6	62.4	60.4	-
G_12_	-	62.8	62.8	62.8	62.8	62.8	62.8	62.8	62.8	62.4	62.2	62.0	-

1– P_0_–phosphorus core; P_1_, P_2_, P_3_, and P_4_—phosphorus atoms of the first, second, third, and fourth generation, respectively.

## Data Availability

All experimental data are provided in the article.

## Sample Availability

Samples of the compounds are not available from the authors.
